# GAIN- BUT NOT LOSS-RELATED VIEWS ON AGING PREDICT MORTALITY OVER A PERIOD OF 23 YEARS

**DOI:** 10.1093/geroni/igac059.1676

**Published:** 2022-12-20

**Authors:** Susanne Wurm, Sarah Schaefer

**Affiliations:** University Medicine Greifswald, Greifswald, Mecklenburg-Vorpommern, Germany; Leibniz Institute for Resilience Research, Mainz, Rheinland-Pfalz, Germany

## Abstract

Some 2 decades ago, Levy et al. (2002) published their seminal study on the impact of SPA on mortality over a period of 23 years. Our study aimed at replicating and extending these findings. Based on a large German population-based sample of individuals aged 40+ (N = 2,400), for whom mortality was also documented over 23 years (1996–2019), we investigated the impact of gain- and loss-related SPA and SA on mortality. Data were analyzed with hierarchical Cox proportional hazard regressions. For individuals who perceived aging as ongoing development risk of death was half that of individuals with less gain-related SPA. Viewing aging as associated with physical or social losses could not predict mortality after controlling for covariates (age, gender, education, health-related variables, and psychological variables). Neither could SA predict mortality. The results suggest that mainly gain-related SPA explain differences in mortality and should thus be addressed in intervention studies.

